# Anatomy of four human Argonaute proteins

**DOI:** 10.1093/nar/gkac519

**Published:** 2022-06-23

**Authors:** Kotaro Nakanishi

**Affiliations:** Department of Chemistry and Biochemistry, The Ohio State University, Columbus, OH 43210, USA; Center for RNA Biology, Columbus, OH 43210, USA

## Abstract

MicroRNAs (miRNAs) bind to complementary target RNAs and regulate their gene expression post-transcriptionally. These non-coding regulatory RNAs become functional after loading into Argonaute (AGO) proteins to form the effector complexes. Humans have four AGO proteins, AGO1, AGO2, AGO3 and AGO4, which share a high sequence identity. Since most miRNAs are found across the four AGOs, it has been thought that they work redundantly, and AGO2 has been heavily studied as the exemplified human paralog. Nevertheless, an increasing number of studies have found that the other paralogs play unique roles in various biological processes and diseases. In the last decade, the structural study of the four AGOs has provided the field with solid structural bases. This review exploits the completed structural catalog to describe common features and differences in target specificity across the four AGOs.

## INTRODUCTION

**Figure 1.**
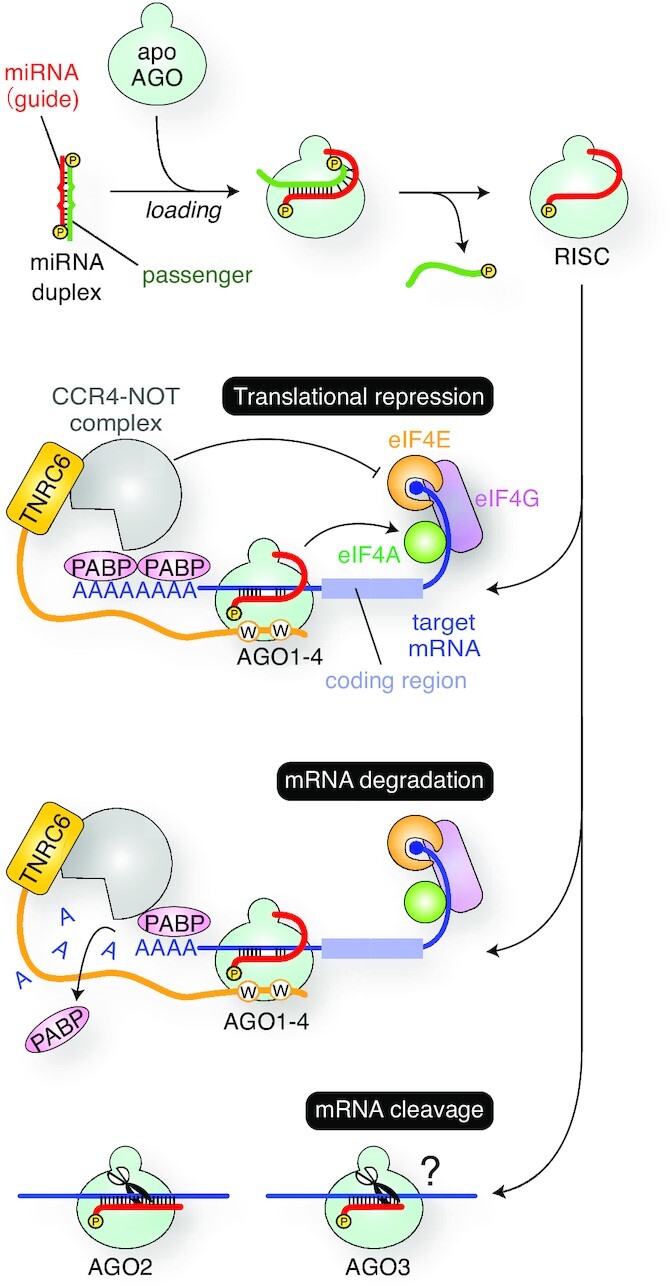
RISC assembly and the gene silencing pathways. AGO incorporates a miRNA duplex, ejects the passenger strand (green), and forms the RISC with the remaining guide strand (red). The effector complexes of all four human AGOs cause translational repression and mRNA degradation. Meanwhile, only AGO2 and AGO3 become a slicer, though target RNAs cleaved by AGO3-RISC remain unknown.

MicroRNAs (miRNAs) are small regulatory RNAs that control gene expression by inhibiting translation or degrading messenger RNAs (mRNAs) when the target mRNAs contain a complementary sequence. In humans, miRNAs are loaded as duplexes into four Argonaute (AGO) proteins to form RNA-induced silencing complexes (RISCs) (Figure 1) (see a previous review (1)). The finding that the four AGOs share ∼80% amino acid identity (2) and about 75% of their bound miRNAs suite (3) inspired the notion that the four AGOs target the same set of RNAs redundantly. Among the four AGO paralogs, AGO2 has been heavily studied as the representative paralog for several reasons: it is ubiquitously expressed as the most abundant AGO in many different types of cells (4), the only essential gene among the four paralogs (5), and was thought to be the only slicer (6, 7).

Previous studies reported that while the four AGO paralogs have largely overlapping functions, each has discrete roles in different cells or under certain conditions when loaded with specific miRNAs (8–10). In addition, gene deletions of AGO1 and AGO3 have been found in patients with neurological disorders (11, 12). These facts indicate that the genes of AGO1, AGO3 and AGO4 are not essential, but their mutation could cause non-lethal but severe diseases.

In the last decade, crystal structures of the four human AGOs in complex with guide RNA were determined (13–18), which revealed how they recognize guide RNAs and provided a solid foundation to discuss their different target specificities. This review will introduce recent updates on the four AGOs, especially the unique roles of AGO1, AGO3 and AGO4. Contradictions in the current consensus and remaining open questions also will be discussed.

## NUMBERING SYSTEM OF GUIDE AND TARGET RNAs

**Figure 2.**
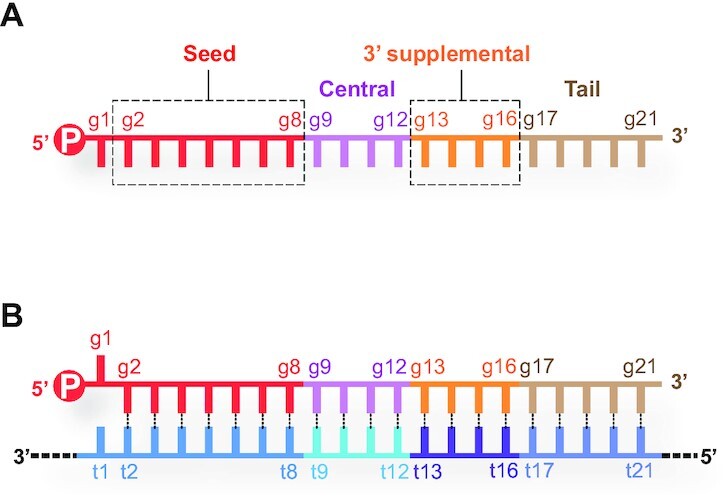
Numbering system of nucleotides on guide and target strands. (**A**) A guide RNA can be split into four regions, seed (red), central (magenta), 3′ supplementary (orange) and tail (wheat). (**B**) Nucleotides on target RNAs (bottom strand) are numbered based on the paired nucleotide on the guide RNA (top strand).

Once miRNAs and small interfering RNAs (siRNAs) are incorporated into AGO as guide RNAs, each nucleotide is designated to play a specific role depending on its position from the 5′ end. The nucleotide at the 5′ end, called the guide nucleotide 1 (g1), is not involved in the target recognition (19–21). The following four segments of g2–g8, g9–g12, g13–g16 and the rest (i.e. g17∼) are referred to as ‘seed,’ ‘central’ ‘3′ supplementary’ and ‘tail’ regions, respectively (Figure 2A). Nucleotides on the target strand are numbered based on their paired guide nucleotide. For example, a nucleotide on the target strand paired with g2 is called target nucleotide 2 (t2) (Figure 2B).

## ARCHITECTURE OF AGO

Here, the domains, subdomains, and loops of AGO will be defined for the purpose of this review.

### Beam runs through the N, L1, L2 and PIWI domains

**Figure 3.**
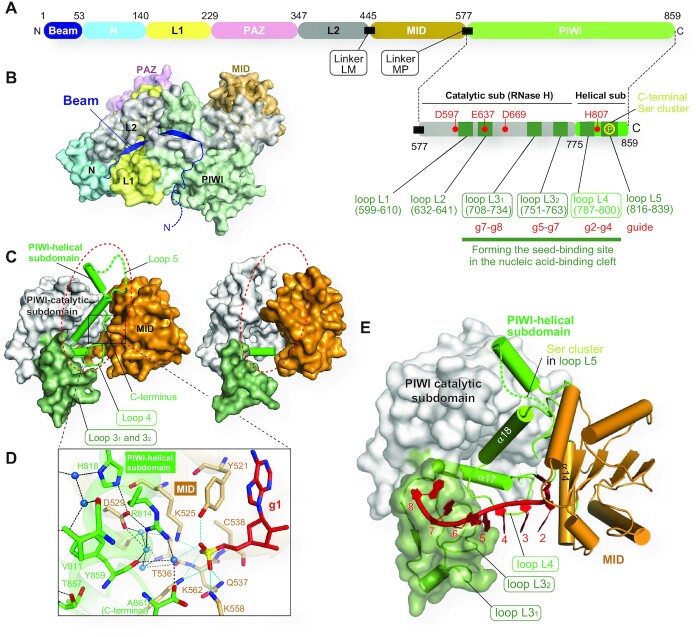
Update on the AGO structure. **(A**) Domain architecture of human AGO. The residue numbers are those of AGO2. Six Loops (dark green) in the two PlWI subdomains are shown on the lower right side. (**B**) Beam running along the bottom of AGO. The color code is the same in Panel A. (**C**) PIWI-helical subdomain (ribbon model) mediates the MID domain (wheat) and PIWI-catalytic subdomain (white) in human AGO4 (left, PDB ID: 6OON). The disorder of a PIWI-helical subdomain of *T. thermophilus* AGO is highlighted with the red dotted circle (right, PDB ID: 3DLB). (**D**) Recognition of the 5′ monophosphate group by the AGO4 MID domain and PIWI-helical subdomain. All residues shown as stick models here are conserved across the four human AGOs. Water molecules are depicted as blue spheres. (**E**) Three Loops of the PIWI domain forming the seed-binding site. The C-terminal serine cluster is located on Loop 5.

It has often been introduced that AGOs consist of four domains: N, PAZ, MID and PIWI (Figure 3A). The N domain serves as a wedge to split duplexes during the RISC assembly (22). The MID and PAZ domains recognize the 5′ and 3′ ends of the guide, respectively (23–25). The PIWI domain is responsible for target cleavage (7, 26, 27). These four domains possess noticeable functions relevant to AGO’s physiological roles. In contrast, the L1 and L2 linkers between the N and PAZ domains and between the PAZ and MID domains, respectively (28), contribute to the structural stability of the RISC. Since the crystal structures revealed that the two linkers are structured (Figure 3B), this review will treat the L1 and L2 as domains. Thus, AGOs are composed of six domains, N, L1, PAZ, L2, MID and PIWI. The N-terminal region preceding the N domain (a part colored in dark blue in Figures 3A and 3B) does not fold into a particular structure. Although the N-terminal half of this region is disordered, the rest shows the continuous electron density map in all crystal structures of human AGOs (13–18). Since the ordered region runs across and reinforces the bottom of AGO by interacting with the N, L1, L2 and PIWI domains, the part is hereafter referred to as ‘Beam’ (Figures 3A and 3B).

### Two PIWI subdomains

The PIWI domain was thought to be a single entity. However, our careful comparison of the guide-bound human AGOs (i.e. RISC) and the guide-free *Neurospora crassa* QDE-2 protein, an AGO homolog, indicated that the PIWI domain could be split into two subdomains (15). One of them, including an RNase H fold, is named the PIWI-catalytic subdomain, while the other, composed of helices, is called the PIWI-helical subdomain (Figures 3A, and 3C left). In addition, the crystal structure of *Thermus thermophilus* AGO, whose 10-nt guide was recognized at its 3′ end by the PAZ domain but did not reach the MID domain due to its short length, had the PIWI-helical subdomain completely disordered (29). As a result, the PIWI-catalytic subdomain and the MID domain physically interacted (dotted red circle in Figure 3C, right). These observations suggest that the two subdomains and the MID domain move independently until the AGO incorporates a guide RNA to form the RISC. The crystal structures also revealed that the PIWI-helical subdomain and the MID domain form a composite binding site for the 5′ monophosphate of the guide RNA (Figure 3D) (15).

### Six loops protruding from the PIWI domain

The PIWI domain has six loops, here named Loops 1, 2, 3_1_, 3_2_, 4 and 5 (Figure 3A and Table 1), all of which are located along the nucleic acid-binding cleft. Loops 1, 3_2_ and 4 have their sequence conserved across the four human AGOs, and part of their Loop 3_1_s are also identical. The role of Loop 1 remains unclear. In contrast, Loop 4, 3_2_, and the conserved region of Loop 3_1_ shape the seed-contact area which directly recognizes the phosphate backbone of the guide RNA at g2–g4, g5–g7 and g7–g8, respectively (Figures 3A and 3E). Loop 2 varies between the four AGOs but retains the catalytic glutamate residue, also known as 'glutamate finger,' that is conserved even in catalytically inactive AGO1 and AGO4 as well as catalytically active AGO2 and AGO3. Loop 2 is thought to have a conformational change upon guide loading which rearranges the glutamate finger to complete the catalytic tetrad (30). Note that the PIWI-helical subdomain includes the fourth catalytic residue (Figure 3A). Given that Loop 2, on which the second catalytic residue is located, moves upon the guide loading, two out of the four catalytic residues converge to complete the catalytic tetrad during RISC assembly (15). This mechanism avoids any promiscuous RNA cleavage by guide-free AGOs. Loop 5 includes a serine-rich region, called the C-terminal serine cluster, where four serine residues are phosphorylated to regulate the affinity of the RISC for mRNAs (31).

**Table 1.** Sequences of six loops in the PIWI domain

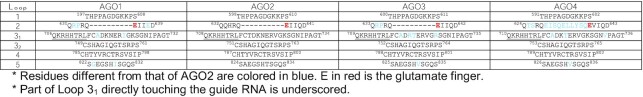

## RISC ASSEMBLY

### A composite seed-binding site

**Figure 4.**
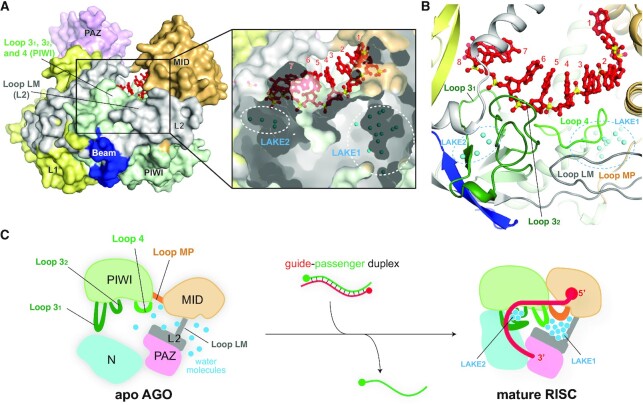
Water molecules underpinning the seed-binding site. (**A**) Surface model (left) and cross-section (right) of human AGO4 in complex with guide RNA (red) (PDB ID: 6OON). Water molecules (blue spheres) forming LAKE1 and LAKE2 are buried inside the AGO4 (right). (**B**) The loops forming the seed-binding site are glued together by LAKE1 and LAKE2. The domain color codes are the same in Figure 3B. The trapped water molecules are depicted as cyan spheres. (**C**) Model of the water-mediated RISC assembly.

Recently, the 1.9 Å resolution crystal structure of AGO4-RISC unveiled two water pouches, referred to as LAKE1 and LAKE2, trapping 13 and 4 water molecules, respectively, inside the protein (Figure 4A) (15). The corresponding water molecules were also found in the crystal structures of other human AGO-RISCs (15). The trapped water molecules are surrounded by five Loops 3_1_, 3_2_, 4, LM and MP, forming the seed-binding site (Figures 3A and 4B). The first three loops stick out from the core of the PIWI domain, while loops LM and MP are linkers between the L2 and MID domains and the MID and PIWI domains, respectively (Figures 3A and 4C). These five loops are connected through water molecules, which work as glue to seal the L2, MID and PIWI domains (Figure 4B). It is remarkable that only the five loops, but neither any α-helix nor any β-sheet, are involved in shaping the seed-binding site. How do AGOs trap these water molecules and why? According to the principle of the hydrophobic effect, water molecules should be excluded from the inside to form a hydrophobic core during protein folding. The simplest explanation would be that, before RISC assembly, each domain of the AGO already folds into a particular structure while its domain linker and loops are free to move around (Figure 4C). This means that, unlike RISC, apo-form AGO does not take a bilobed structure yet and the abovementioned five loops must be unstructured and exposed to the solvent. Once the MID domain and the PIWI-helical subdomain capture the 5′ end of the guide, Loops 3_1_, 3_2_ and 4 would recognize the phosphate backbone of the seed region. Together with LM and MP linkers, these loops must trap solvent water molecules to neutralize their charged and polar parts. The existence of two water clusters, LAKE1 and LAKE2, seems to be evidence that RISC assembly accompanies drastic conformational changes.

### Sorting of small RNAs

In flies, Ago1 and Ago2 use specialized components to selectively load miRNAs and siRNAs, respectively (32, 33). In contrast, humans do not have such a sorting system, and thus it has been thought that small RNAs are randomly incorporated into the four AGOs based on the miRNA populations in the cell (3, 34, 35). Supporting this idea, RNA-sequencing (RNA-seq) of AGO-associated small RNAs showed that most guide RNAs are found across all human AGO paralogs. For example, AGO2- and AGO3-associated small RNAs showed a large overlap (36). Meanwhile, this study reported that AGO2 bound to miR-342-3p 19-fold more than AGO3, whereas AGO3 was associated with miR-629-3p and miR-92b-3p 15- and 12-fold, respectively, more than AGO2. Similarly, miRNA biases were seen also between AGO1 and AGO2 (37). Another study showed that in L591 cells, derived from EBV-positive Hodgkin’s lymphoma cells, non-miRNAs are found more often in AGO1 than in AGO2 (38). Alu element-derived repeat-inducing RNAs are loaded into AGO3 to initiate neural development (8). miR-3191-5p is found in only AGO2 and AGO4 (9). Like mirtrons (39), Agotrons are 80–100 nt short intron-derived regulatory RNAs, but they bypass Dicer1-processing and are loaded into AGO1 and AGO2 (40). Agotrons use their 5′ end 20 nt to repress the gene expression including a complementary sequence. However, the secondary structure prediction of Agotrons, Mast1 and Pkd, indicates that their 5′ end 20 nt are GC-rich and form a stable stem-loop structure, likely occluding the 5′ end to be captured by the AGO. These results strongly suggest that there are yet-unidentified systems that enable specific AGO(s) to load a subgroup of small RNAs. Further studies will be required to understand whether each AGO possesses an ability to sort specific small non-coding RNAs autonomously or needs to interact with components to discriminate a subgroup of miRNAs.

### Asymmetric guide selection

**Figure 5.**
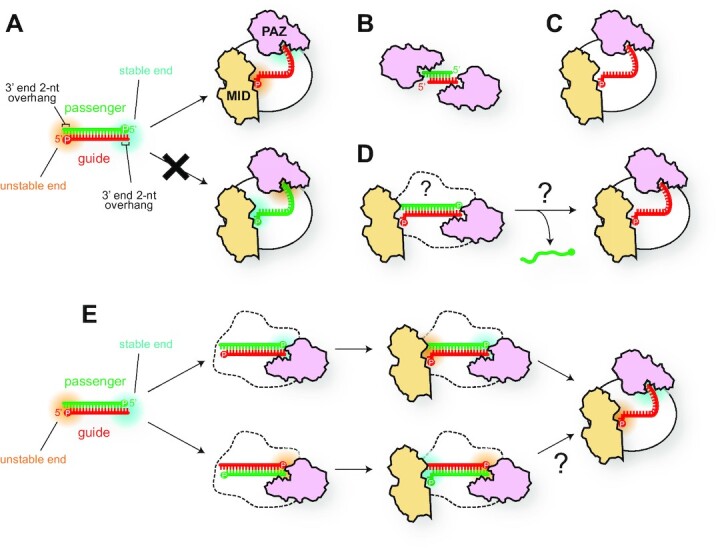
Asymmetric guide selection. (**A**) A strand with a thermodynamically unstable 5′ end (red strand) is incorporated as the guide into AGO. The thermodynamically stable and unstable ends are highlighted with cyan and orange blurs, respectively. The MID and PAZ domains are colored in wheat and pink, respectively. A 5′ monophosphate group is shown as ‘P’ in a circle. (B, C) Schematics of the crystal structures of PAZ-duplex complex (**B**) (23) and *T. thermophilus* AGO in complex with guide RNA (**C**) (29). (**D**) The currently accepted mechanism of asymmetric guide selection. The overall structure of AGO during RISC assembly remains unclear. (**E**) The PAZ domain binds to either of the duplex termini, resulting in the binding of the MID domain to the other end. In the bottom pathway, when the PAZ domain binds to the thermodynamically unstable end (the 3′ end of the green strand), it remains unclear how the MID domain can recognize the 5′ end of the red strand and take the strand as a guide.

It is well known that the guide-strand selection by AGOs follow two rules. The thermodynamic rule is that the thermodynamically unstable end of the miRNA- and siRNA duplexes is frayed, and the strand whose 5′ end is captured by the MID domain is loaded as the guide strand into AGO to form the RISC (Figure 5A) (41, 42). Another rule is the 5′ base preference which the MID domain exploits with its nucleotide-specificity loop to preferentially recognize adenine or uracil of the nucleotide at the g1 over cytosine or guanine (43). Although several models have been proposed, we do not seem to have an ideal mechanism that can explain both rules adequately without contradiction.

After Dicer crops the loop of pre-miRNAs, the resultant double-stranded RNAs are licensed to serve as miRNA duplexes with the two hallmarks: a monophosphate group at each recessed 5′ end and a 2-nt overhang at each 3′ end (Figure 5A). The significance of these two structural features is validated by a series of biochemical and structural studies. The 5′ monophosphate group serves as the hub of the hydrogen bond network between the MID and PIWI domains (Figure 3D) (24, 25). Therefore, the 5′ monophosphate group of guide strands is essential for the RISC assembly of eukaryotic AGOs. On the other hand, the significance of the 3′ end 2-nt overhang for duplex loading is evidenced by RNA-binding assays using small RNA hairpins with a blunt end as competitors (44). This significance was validated by the crystal structure of the isolated human AGO1 PAZ domain in a complex with a siRNA-like duplex (Figure 5B) (23). The structure showed two PAZ domains bound at both 2-nt overhangs of the duplex. After that, the first crystal structure of the RISC from *T. thermophilus* revealed that the PAZ domain captures the 3′ end of the single-stranded guide DNA, while the MID domain recognizes the 5′ end (Figure 5C) (29). These two structures have been strong grounds for believing the model that the MID and PAZ domains capture the 5′ and 3′ ends, respectively, of ‘the guide strand’ when the AGO loads a duplex, though the structural basis remains elusive (Figure 5D).

A previous study quantified the affinity of the PAZ domain for the 3′ end 2-nt overhangs to be 0.9–2.0 nM (23). In contrast, the isolated MID domain has a dissociation constant of 0.1–3.6 mM for free nucleoside monophosphates that mimic the 5′ end nucleotide of guide RNA (i.e. nucleotide at g1) (43). These results indicate that the affinity of the PAZ domain for the 3′ end 2-nt overhang is 5 × 10^4^- to 4 × 10^6^-fold higher than that of the MID domain for the 5′ end nucleotide. Therefore, it is reasonable that binding of the PAZ domain to miRNA duplexes is a prerequisite for that of the MID domain. The nucleotide specificity, however, resides only in the MID domain (43). The broadly accepted model mechanism is that the PAZ domain captures the more stable end while the MID domain binds the end that is less thermodynamically stable (Figure 5E, top) (45, 46). However, if the PAZ domain binds to the unstable end of the duplex (Figure 5E, bottom), the MID domain can only recognize the stable 5′ end, which contradicts the thermodynamic rule. Indeed, a previous study incubated the isolated AGO2 PAZ domain with an siRNA duplex having zero, one, or two nucleotide mismatches at both ends and revealed that the PAZ domain preferentially binds to a more thermodynamically unstable end (42). The authors also reported that a PAZ-deleted AGO2 mutant retained the asymmetric guide selection and suggested that the PAZ domain is not necessarily required for the guide strand selection (42). The Kay group reported that the PAZ domain is indispensable for the RISC assembly of slicer-competent AGO2 because it can cleave the passenger strand. However, slicer-deficient AGO1, AGO3 and AGO4 still require the PAZ domain to assemble their RISC (47). Supporting this idea, the Shin group suggested that the PAZ domain vigorously shakes the guide 3′ end to eject the passenger strand during RISC assembly (48). These results indicate that the AGO PAZ domain seems to play a critical role in the passenger ejection, albeit it would contribute to guide selection to some extent because passenger ejection is the last step of guide selection. Further studies will be required to elucidate the molecular basis for asymmetric guide selection.

### Arm switching

**Figure 6.**
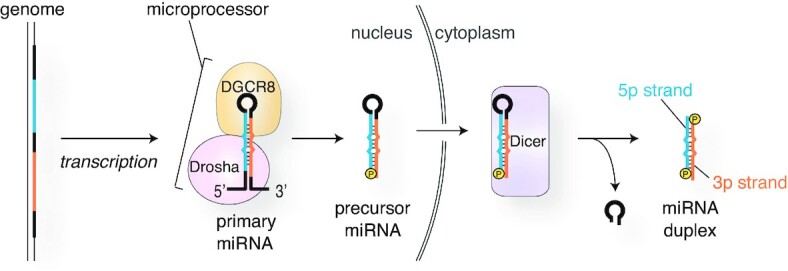
miRNA processing pathway.

**Figure 7.**
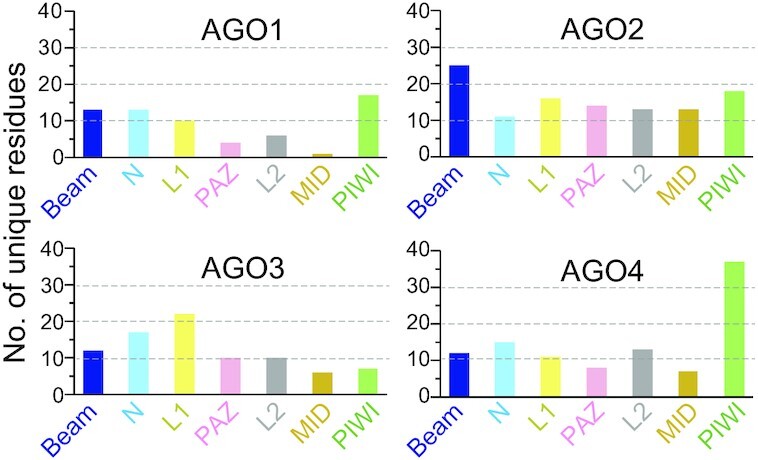
The number of residues unique to each AGO per domain.

In humans, the genes of miRNAs are transcribed by mainly RNA polymerase II into primary miRNAs and processed by Microprocessor complex composed of a complex of Drosha, a nuclease of the RNase III family, and its binding protein DGCR8 (Figure 6) (49–52). The loop of the product called precursor miRNAs (pre-miRNAs) is cropped by Dicer, another RNase III enzyme (53). The resultant miRNA duplexes consist of the 5p and 3p strands, which are derived from the upstream and downstream regions of the cropped loop in their pre-miRNAs (Figure 6). The miRNA duplex of let-7a is composed of let-7a-5p and let-7a-3p. Both strands are loaded into AGO1, AGO2 and AGO4 at almost equal efficiencies, but AGO3 preferentially loads let-7a-3p over the 5p strand (54). Domain-swapping experiments showed that any chimeric constructs had a higher affinity for let-7a-3p over the 5p if they retained AGO3 PAZ, L2 and MID domains. AGO3-specific loading preference must reside in these regions. Interestingly, none of these domains has many residues unique to AGO3 (Figure 7).

## TARGET SPECIFICITY OF RISC

The target specificity of miRNAs has been discussed thoroughly in the context of nucleic acids, such as the sequence complementarity with target sites, adenine at t1, the location of target sites within the 3′ UTR, the secondary structures around the target site, and so on (21, 55, 56). However, the experimental data show that the query miRNA does not always repress the expression of the predicted target genes, even though their sequences are highly complementary (57–59). The facts indicate that, to understand the underlying molecular mechanism of target specificity, we need to take account of more aspects, which will be discussed in this section.

### The proteinaceous part of the RISC contributes to the target specificity

**Figure 8.**
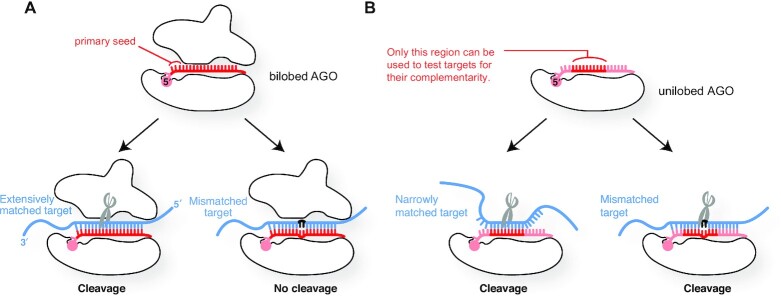
Roles of the N-PAZ lobe. (**A**) The bilobed structure is essential for high target specificity and mismatch recognition. (**B**) The lack of the N-PAZ lobe impairs the target specificity and mismatch recognition.

MiRNAs loaded into AGO do not always follow the rules of binding and dissociation which nucleic acids intrinsically possess, but rather behave as if they are part of an RNA-binding protein (60). This means that the contribution of the proteinaceous part (i.e. the AGO within the RISC) to the target specificity is not negligible. Like human counterparts, yeast *Kluyveromyces polysporus* Ago1 has a bilobed structure composed of the N-PAZ lobe (also known as the N-terminal lobe) and the MID-PIWI lobe (also known as the C-terminal lobe) (30). A bilobed yeast Ago1 construct uses the g2–g23 of a 23 nt guide RNA to check the complementarity with targets and cleaves only RNAs fully base-paired to the guide (Figure 8A) (30, 61). Although an isolated MID-PIWI lobe retains most abilities of AGO, such as duplex loading, passenger ejection, guide recognition, target binding, and target cleavage, the unilobed Ago1 fails to avoid cleaving mismatched targets (discussed later) (61).

The crystal structures showed both unilobed and bilobed AGOs arrange the g2–g7/g8 in an A-form helical structure (30, 61) to increase their affinity for mRNAs up to ∼300-fold (62). Bilobed AGOs solvent-expose the g2–g4, which works as the primary seed, while sequestering the rest of the guide from contacting target mRNAs. In contrast, the crystal structure of the MID-PIWI lobe indicates that the unilobed construct exposes all guide nucleotides to the solvent due to the lack of an N-PAZ lobe (61). As a result, this unilobed construct cannot make the primary seed (g2–g4) and uses only the g5–g14 when checking the complementarity with targets prior to cleavage, lowering its target specificity (Figure 8B). Even worse, the MID-PAZ lobe alone fails to discriminate mismatched targets and cleaves them. These results demonstrate that an N-PAZ lobe and a MID-PIWI lobe work together to create the primary (g2–g4) and secondary (g5–g8) seeds sequentially to maximize the number of guide nucleotides involved in the target recognition, establishing the high target specificity of the RISC. Thus, the two lobes not only shape the guide binding site but also actively participate in target recognition.

### The nucleic acid-binding cleft

A series of studies completed the structural catalog of the four human AGO-RISCs and revealed their common and unique features (13–18).

#### Y-shaped guide-binding cleft

**Figure 9.**
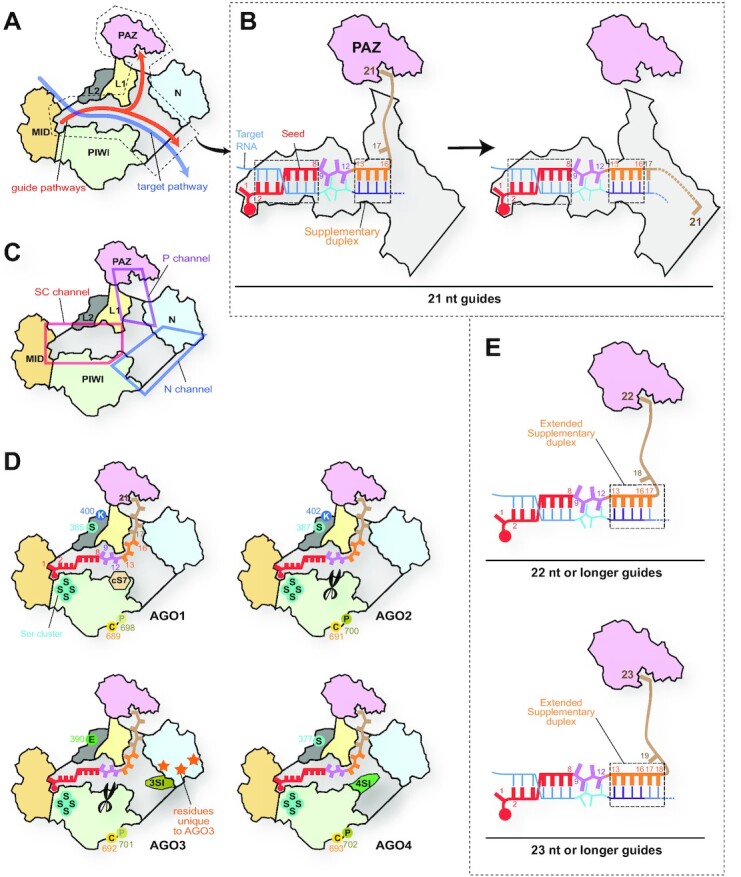
Differences in the N channel between four human AGOs. (**A**) Pathways of guide and target strands. The domain color codes are the same as in Figure 3. The nucleic acid-binding cleft is colored in gray. (**B**) Transfer of the guide 3′ supplementary and tail regions between two branched channels. In the case of a 21 nt guide, the g2–g8 and g13–g16 serve as the target binding sites. (**C**) Y-shaped nucleic acid-binding cleft. The cleft is composed of the Seed-Central channel (SC channel in the red box), the PAZ channel (P channel in the magenta box), and the N channel (blue box). (**D**) Local structures unique to each AGO. cS7, 3SI and 4SI are localized in the N channel. The catalytic DEDH tetrad is depicted as scissors. AGO3-specific residues on its N domain are shown as orange stars. The residues undergoing post-translational modification are shown with their residue number (see Figure 10). (**E**) Model of expanding the 3′ supplementary region. When the guide lengths are 22 nt (top) and 23 nt (bottom), the g17 and the g17–g18 participate in target recognition as part of the 3′ supplementary region, respectively.

The previously determined crystal structures of human RISCs show the trajectories of the bound guide RNA (Figures 9A and 9B) (13–18, 63, 64). The seed and central regions of the guide (i.e. g2–g12) always occupy a space between the L1/L2 and PIWI domains. This space is hereafter called the ‘Seed-Central channel (SC channel)’ (Figure 9C). Meanwhile, the rest of the guide (i.e. the 3′ supplementary and tail regions) move between two branched channels (Figures 9A and 9B). When the guide 3′ end is anchored at the PAZ domain, the 3′ supplementary and tail regions pass through a channel between the N and L1 domains. This intervening space is named the ‘PAZ channel (P channel)’ (Figure 9C). Once the guide 3′ end is released from the PAZ domain, the 3′ supplementary and tail regions move to the other branched channel between the N and PIWI domains, which is named the ‘N channel’ (Figures 9B and 9C). In contrast, target RNAs have a single pathway from the SC to the N channel (Figure 9A).

#### Guide RNAs recognition by AGO in the absence of target RNA

**Figure 10.**
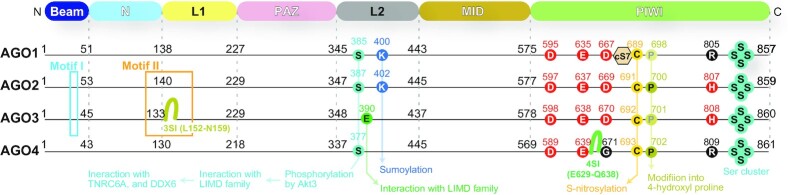
Locations of the motifs, insertions, and modification sites. Red circles are the catalytic tetrad. Numbers in black indicate the first residue on each domain.

Most crystal structures of target-free RISCs showed a continuous electron density map of the g1–g7 (or g8), whereas the density map of the guide after the g8 is very poor or completely disordered (13–18). The continuous density map of the tail region running through the P channel was seen in only a few RISC structures (15, 65). These observations indicated that AGO does not recognize all the guide nucleotides in the RISC. For example, in the case of a 21-nt guide RNA, the seed (g1–g8) and the g21 are anchored along the SC channel and at the PAZ domain, respectively, while the central (g9–g12), 3′ supplementary (g13–g16), and tail regions (g17–g20) are free to move within the SC and P channels (Figure 9A). The electrostatic potentials showed a positively charged area running across the SC to the P channel (Supplementary Movie 1). Notably, the amino acid residues forming this guide-contacting area are identical among the four AGOs. On the other hand, AGO1, AGO3 and AGO4 have unique local structures, such as the conserved segment 7 (cS7), AGO3-Specific Insertion (3SI), and AGO4-Specific Insertion (4SI), respectively (Figures 9D and 10), but none of them, except for 4SI, seems to be involved in the guide recognition unless the RISC binds to target RNAs. Therefore, at least AGO1, AGO2 and AGO3 recognize the bound guide RNAs in the same manner when the PAZ domain captures the guide 3′ end.

The crystal structure of AGO2 showed that the seed and 3′ supplementary regions paired with the target and that the guide was kinked just after g16 to direct the tail region towards the P channel (Figure 9B, left) (64). Once the g17 becomes involved in pairing with target RNAs, the guide 3′ end is released from the PAZ domain, which moves the tail region from the P channel to the N channel (Figure 9B, right) (63). When the guide length is 22 or 23 nt, AGOs can likely use the g17 or the g17–g18 as part of the 3′ supplementary region while the guide 3′ end remains bound to the PAZ domain (Figure 9E). Thus, long guide RNAs would have an extended 3′ supplementary region for target inspection.

#### N channel is the hot spot of local structures unique to each AGO

Whereas four human AGOs share the same guide-contacting area, each paralog possesses unique local structures that make their target specificity different when the guide 3′ supplementary region recognizes target RNAs. The N channel, consisting of the N, L1 and PIWI domains, includes many residues unique to each AGO. This section will describe the specific local structures found in the N channel of AGO1, AGO3 and AGO4.

##### AGO1

The nucleic acid-binding clefts of AGO1 and AGO2 have the same shape because each of their domains is composed of the same number of residues without any insertion or deletion (Figure 10). The only main difference in their nucleic acid-binding cleft is the cS7 unique to AGO1 (Figures 9D and 10) (13, 18). The crystal structure of AGO1-RISC suggested that the cS7 would work as a steric hindrance to prevent the target strand from being arranged in the vicinity of the pseudo-catalytic DEHR tetrad. However, swapping the cS7 alone with the counterpart of AGO2 did not confer slicing activity onto AGO1 (13), suggesting that the slicer deficiency of AGO1 is not due to the existence of cS7. On the other hand, when only the fourth residue, Arg805, was replaced with histidine (i.e. switching DEDR to DEDH), the AGO1 mutant showed weak activity, which was enhanced by replacing the cS7 with the counterpart of AGO2 (13). These results demonstrate that AGO1’s slicer deficiency is attributed to the pseudo-catalytic DEDR tetrad and that having the cS7 changes the way of target recognition from that of AGO2. This distinct target recognition may be specialized for AGO1-specific role(s) that we have not known yet.

##### AGO3

AGO3 has two major differences in the nucleic acid-binding cleft from AGO2 (Figures 9D and 10) (14, 66). First, the AGO3 L1 domain possesses the 3SI encompassing L152-N159. In the crystal structure of AGO3-RISC, a loop of 13 amino acid residues, including the 3SI, was disordered but seemed to be able to interact with the guide 3′ supplementary region (g13–g16/g17) and the target (t13–t16/t17) in the N channel once the guide 3′ end was released from the PAZ domain (14). Second, the N domain has 13 residues unique to AGO3 on the surface near the 3SI (14). These structural features make AGO3′s N channel quite different from that of the other paralogs and would enable AGO3 to be catalytically activated by specific tiny RNAs (tyRNAs) (which will be discussed later) (67).

##### AGO4

The AGO4 PIWI domain is furnished with 4SI, corresponding to E629-Q638 on Loop 2 (Figures 9D and 10) (15, 68). The crystal structure of AGO4-RISC indicates that the 4SI sticks out into the N channel (15). A docking model suggested that the 4SI could interact with the guide and target strands, whereas the counterpart of AGO2 has a kink turn that is too short to do so. A 4SI-depleted AGO4 mutant bound to the target RNA more efficiently than the wild type, suggesting that the 4SI is involved in target recognition and makes AGO4′s N channel less accessible to targets.

**Figure 11.**
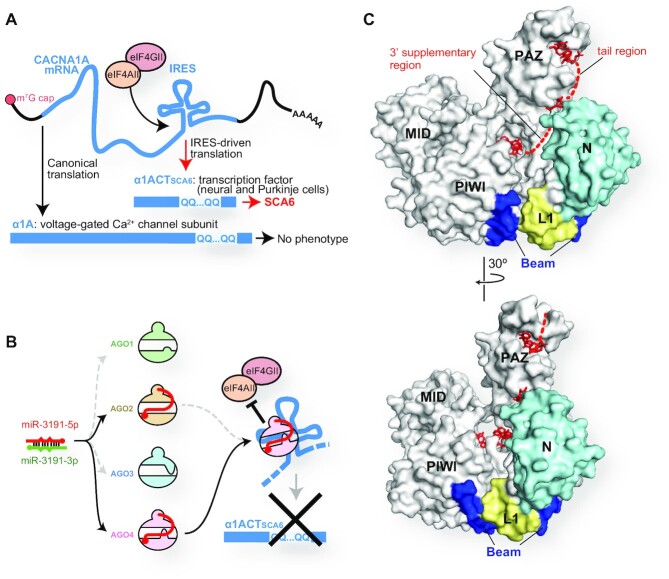
Unique roles of AGO4. (**A**) IRES-driven translation of CACNA1A mRNA causes spinocerebellar ataxia 6 when the mRNA includes polyglutamine repeats. (**B**) Both AGO2 and AGO4 load miR-3191–5p, but only the AGO4-RISC stops the IRES-driven translation. (**C**) The N-terminal regions of AGO4 highlighted in cyan, yellow and blue serve as the DNMT3A-binding site. The bound guide RNAs are depicted as red stick models (PDB ID: 6OON).

On the other hand, AGO4 is known to have a specialized target specificity when loaded with specific miRNAs. For instance, AGO4 loaded with miR-3191–5p can bind to *CACNA1A*, a bicistronic mRNA with an internal ribosome entry site (IRES) (69). The canonical translation requiring the 5′-end cap produces a voltage-gated calcium channel subunit, α1A from the CACNA1A mRNA, while IRES-driven translation generates α1ACT, which serves as a transcription factor essential for the growth of neurons and Purkinje cells (Figure 11A) (69). A mutation of polyglutamine repeats in the C-terminal region of α1A does not affect its function, whereas the same mutation on the α1ACT causes spinocerebellar ataxia type 6 (69–72). The IRES-driven translation is triggered by the binding of eIF4AII and eIF4GII (Figure 11A). Both AGO2 and AGO4 incorporated miR-3191–5p, but only the AGO4-RISC bound the IRES to inhibit the recruitment of the two initiation factors (Figure 11B) (9). Presumably, miR-3191-5p cooperates with 4SI to shape a unique target binding site on AGO4. This could explain why AGO2 loaded with miR-3191-5p does not bind to the IRES. Alternatively, a protein recognizing the 4SI may recruit only AGO4 to the IRES.

A previous study reported that siRNA silencing AGO4, but neither AGO1, AGO2 nor AGO3, decreased the methylation level of miRNA-181a-5p (73). This study showed that de novo DNA methyltransferase 3 (DNMT3A) forms a complex with AGO4 to modify the cytosine of miRNAs into 5-methylcytosine (5mC), which inhibits the miRNA functions and is associated with poor prognosis in glioblastoma multiforme (73). An antibody that recognizes the AGO4 N-terminal 164 residues prevented the interaction of AGO4 with DNMT3A, suggesting that the DNMT3A-binding site resides in the first 164 residues of AGO4. This region forms the edge of the N channel and includes 36 out of the 103 AGO4-specific residues (Figures 7 and 11C) (15). Therefore, it is likely that DNMT3A binds to the N channel and modifies cytidine(s) on the accessible 3′ supplementary and tail regions of the AGO4-associated guide RNAs. A previous genome-wide association study reported that depletion of AGO4 resulted in demethylation at the known AGO4-binding loci, *TRDP*, *C16ORF89* and *ATAT1* (74), suggesting that AGO4 is involved in the de novo methylation of DNA. AGO4 could methylate DNA even in the presence of azacitidine, an inhibitor of DNA methyltransferase 1 (DNMT1) which recognizes hemimethylated CpG on the parental strand. The above-mentioned interaction between AGO4 and DNMT3A could also explain how DNMT3A is recruited to and methylates the AGO4-binding genomic loci.

### Positively charged surface of RISCs may be bifunctional

**Figure 12.**
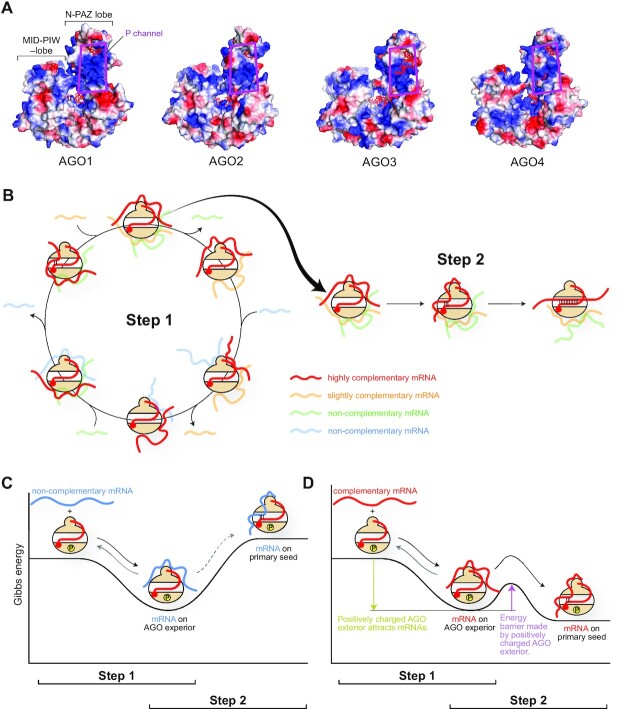
Bifunctional positively charge exterior of AGO. (**A**) Electrostatic potential map of four human AGOs. Positive and negative potentials are drawn in blue and red, respectively. The P channel is highlighted with magenta boxes. (**B**) Model of two-step RNA recognition by AGOs. Slightly- and non-complementary mRNAs are made fainter for clarity. (C, D) Energy levels of non-complementary mRNA (**C**) and complementary mRNA (**D**). AGO is phosphorylated to some extent.

Ameres *et al.* used single-stranded target RNAs whose sequence is the same as the guide and observed that the non-complementary targets weakly interact with the RISC (75). Another study using a single-molecule approach removed major miRNA-binding sites from the reporter gene and still observed that about 10% of the mRNA bound to RISCs (76). These results indicated a guide-independent, non-specific interaction between RISCs and mRNAs. RISCs solvent-expose the primary seed (i.e. g2-g4) to scan the complementary sequence from a pool of mRNAs. It is not realistic that RISCs find the best complementary mRNAs on the first attempt. It is also unrealistic that the first contact of RISC with mRNAs always occurs through the primary seed. Given that RISCs have many positively charged patches on their exterior (Figure 12A) (13–18), it seems reasonable that the effector complexes encounter many weak ‘off-target’ interactions on their exterior until the RISCs eventually meet the best target. I surmise that RISCs achieve the high-throughput target screening using the following two-step RNA recognition (Figure 12B).

The first step is to increase the opportunity to meet as many mRNAs as possible. RISCs must exploit their positively charged patches to interact with the negatively charged phosphate backbone of mRNAs. This step is a sequence-independent, ionic interaction. Since the positively charged patches are scattered on the surface, RISCs seem to bind to multiple regions on the same or different mRNAs simultaneously. The four human AGOs use the same strategy in the first step of their target screening. But presumably, their affinity for mRNAs would vary due to the difference in their electrostatic potential maps (Figure 12A). The exterior charge should be positive enough to attract mRNAs but not too strong to release bound ‘off-target’ mRNAs (Figure 12C). As a result, the dwelling time of a bound mRNA would be short, unless part of the RNA is base paired with the primary seed. After releasing such ‘off-target’ mRNAs, the positively charged patches would become accessible to other mRNAs in the pool (Figure 12B, left). This circulation must increase the population of the target screening. The dwelling time of mRNAs bound to the positively charged patches on the AGO exterior would be extended if the bound mRNAs start pairing with the primary seed, which is the second step of target screening (Figure 12B, right). The positively charged patches on the RISC play a different role from the first step. These patches could compete with the primary seed for the transiently bound mRNAs. Thus, the positively charged patches would work as an energy barrier to achieve ‘on-target’ interaction in the second step (Figure 12D). When the sequences of the guide and target are complementary, the SC channel becomes wider to render the secondary seed (i.e. g5-g7/g8) accessible to the target to further test their complementarity (see the review paper (77)). It is plausible that the four human AGOs set unique energy barriers to pursue their specific ‘on-target’ interactions, given the different arrangements of positively charged patches on their surface (Figure 12A) and their unique local structures (Figure 9D). Supporting this, a recent study showed that AGO2 and AGO3 recognize the flanking regions of the miRNA-binding site differently (14). However, little is known about how the RISC exterior interacts with mRNA in a guide-independent manner.

### Target RNAs of tyRNAs

The long journey of miRNA biogenesis has been thought to end when their mature form of 19–23 nt is loaded into an AGO to form the RISC (Figures 1 and 6). Meanwhile, although RNA-seq studies have reported 13–18 nt AGO-associated tyRNAs, little is known about the biogenesis of tyRNAs. Most of the tyRNAs are derived from tRNAs (78–80), but some of them are synthesized from miRNAs (81), which may suggest that the final destination of the miRNA biogenesis could be their tyRNAs.

Target-directed miRNA degradation (TDMD) is a phenomenon in which the degradation of AGO-associated miRNAs is induced by extensively complementary mRNAs called TDMD-inducing mRNAs (82–84). Recent studies revealed the molecular mechanism by which the 3′ supplementary and tail regions of AGO-associated miRNAs are paired extensively with TDMD-inducing mRNAs and form a scaffold to recruit a Cullin-RING E3 ubiquitin ligase complex. This complex leads to polyubiquitination of the AGO for 26S proteasome-mediated degradation, which results in exposing miRNAs to the cellular nucleases (85, 86). Since tyRNAs lack the 3′ half of miRNAs that is essential to trigger TDMD, AGOs loaded with tyRNAs may escape from degradation (87). It remains an open question whether tyRNAs recruit AGO to the same set of mRNAs as their parental miRNAs or play different roles in specific cellular events.

## SLICING ACTIVITY

### Which AGOs retain slicing activity?

Among the four human AGOs, AGO2 and AGO3 share the same catalytic DEDH (Asp-Glu-Asp-His) tetrad (Figure 10) (30). Nevertheless, only AGO2 has been shown to have slicer activity when target RNAs are fully complementary to the guide (6, 7). Previously, two groups swapped Motif I in Beam and Motif II between the N and L1 domains (Figure 10), and the resultant AGO3 chimera cleaved RNAs when programmed with full-length miRNAs, like AGO2 (66, 88). This result indicated that the AGO3 PIWI domain retains a slicing activity, which is repressed by the two AGO3 Motifs. After a while, my group discovered that specific full-length miRNAs, such as miR-20a, but not let-7, miR-16 or miR-19b, catalytically activate AGO3, albeit the activity was much lower than that of AGO2 (14). My following study revealed that AGO3 becomes a comparable slicer to AGO2 when loaded with 14 nt tyRNAs of miR-20a, let-7a, miR-17, miR-18a, miR-27a and miR-92a whose 3′ 7–9 nt are deleted (67). However, 14 nt variants of miR-16, miR-19a, or miR-19b did not activate AGO3, indicating that not all 14 nt tyRNAs confer slicing activity on AGO3. Based on these results, tyRNAs capable of catalytically activating AGO3 were named cleavage-inducing tyRNAs (cityRNAs). CityRNAs should be about 14 nt long and have specific sequences, but the sequence requirements for cityRNAs remain unknown. In contrast, AGO2 had significantly decreased slicing activity when loaded with tyRNAs. This finding demonstrates that AGO2 and AGO3 have different optimum lengths of guide RNA for slicing activity and, to the best of my knowledge, is the first example that miRNAs drastically change the roles depending on their length.

How about the slicing activity of the other two paralogs? It is unlikely AGO4 retains slicing activity because the third and fourth catalytic residues are replaced with glycine and arginine, respectively (Figure 10). On the other hand, AGO1 substitutes arginine for only the fourth catalytic histidine residue (Figure 10). A previous study showed that AGO1 cleaved the passenger strand during RISC assembly (89), albeit others reported no slicing activity for AGO1 (13, 18,66). There may be a possibility that yet-unidentified specific guide RNAs can activate AGO1, as in the case of AGO3 (14,67).

### Mechanisms of RNA cleavage by AGO2 and AGO3

**Figure 13.**
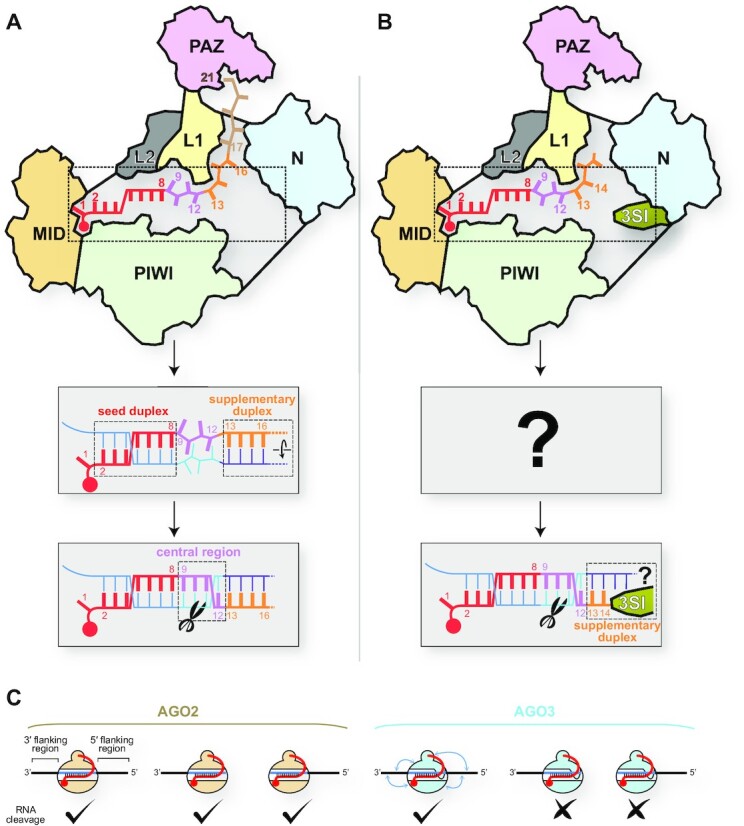
Differences in target recognition between AGO2 and AGO3. (**A**) Model of target cleavage by AGO2 loaded with a full-length guide. (**B**) Model of target cleavage by AGO3 loaded with cityRNA. (**C**) Flanking regions of the miRNA-binding site are required for AGO3, but not AGO2, to cleave target RNAs when they are programmed with full-length miRNAs.

A model of target cleavage by AGO2 was recently proposed based on the previously reported biochemical and structural data (90). First, target strands are primarily paired with the seed region of the AGO2-bound guide RNA (g2–g8) and subsequently with the 3′ supplementary region (g13–g16) (Figure 13A). In this step, the target is not yet paired with the central region of the guide (g9–g12). Second, only when both the seed- and 3′ supplementary-duplex segments are perfectly complementary, the latter segment is twisted to let the central region form a duplex with the target strand (Figure 13A bottom). To rotate only the 3′ supplementary duplex segment, AGOs anchor the phosphate backbone of the seed region, but not the central or the 3′ supplementary regions. Finally, AGO2 cleaves the target strand between t10 and t11 if the central region is also fully paired. This model was visualized by the recent crystal structure of the ternary complex of AGO2 with a 21-nt guide RNA and a perfectly complementary target RNA (64). Although an AGO2 catalytic mutant was employed in this study to avoid target cleavage during crystallization, the central region was not paired. Instead, the seed and 3′ supplementary regions form duplex segments. These observations are consistent with the proposed model.

This AGO2 activation mechanism does not seem to be applied to RNA cleavage by cityRNA-loaded AGO3 because the 3′ supplementary region of 14 nt guides consists of only two nucleotides (i.e. g13–g14), which is too short to form a stable duplex segment (Figure 13B). In addition, AGO3 activation heavily depends on the guide sequence, unlike AGO2. Therefore, AGO3 must use a different mechanism to recognize and cleave target RNAs. Since the 3SI protrudes into the N channel, the unique local structure may cooperate with cityRNAs to cleave target RNAs.

The requirements for AGO3 activation are beyond the guide RNAs. AGO3 loaded with a 23-nt miR-20a drastically reduced slicing activity when the target site lacked the 5′ or 3′ flanking region (Figure 13C, right) (14). This result suggests that both flanking regions are essential for sufficient target cleavage by AGO3. In contrast, AGO2 loaded with the 23-nt miR-20a cleaved the target RNAs even without the 5′ or 3′ flanking region (Figure 13C, left). These observations suggest that at least AGO3 recognizes the flanking regions of miRNA-binding sites, presumably due to its unique electrostatic potential surface, as discussed earlier.

### Eukaryotic AGOs do not cleave DNAs

Prokaryotic AGOs use DNA, RNA, or both as their guide to cleave target DNAs, RNAs or both, and their guide-target combinations vary from species to species (91–93). Given that eukaryotic AGOs evolved from prokaryotic AGOs, it is not surprising that they can also bind to promoter and enhancer regions to control gene expression (94–96). Although eukaryotic AGOs physiologically load only RNA guides, human AGO2 and yeast Ago1 programmed with DNA guides cleave RNA but not DNA targets in vitro (97, 98). These results may indicate that eukaryotic AGOs intrinsically lack DNase activity because otherwise, they could cleave DNA and disrupt the genome integrity. Therefore, it seems reasonable that eukaryotic AGOs may have had to lose the ability to cleave DNA during their molecular evolution to control transcriptional gene silencing (TGS). Thus far, little is known about the mechanism by which eukaryotic AGOs avoid cleaving DNAs. However, it also may be possible that eukaryotic AGOs earn a deoxyribonuclease activity under special conditions, given that the C-terminal fragment of *Caenorhabditis elegans* Dicer became a DNase upon proteolytic cleavage (99).

## TNRC6 PROTEIN (GW182)

TNRC6 proteins bind preferentially to RISCs over guide-free AGOs (100, 101), indicating that only mature RISCs ready to participate in gene silencing are allowed to bind to TNRC6 proteins. Comparing the conformations of apo-form AGO and RISC is critical to understanding the molecular mechanism of the RISC-TNRC6 interaction, which has been hampered by a lack of structural information of apo-form human AGO. Meanwhile, accumulated data suggest that the two PIWI subdomains and the MID domain converge during the RISC assembly (15). Therefore, it is likely that their convergence completes the TNRC6-binding site on the RISC. This model could explain how AGOs earn their high affinity for TNRC6 proteins only after RISC assembly (100, 101).

**Figure 14.**
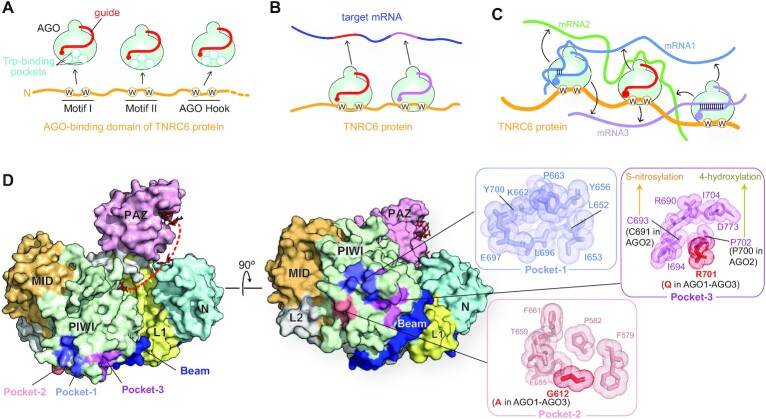
Interaction between RISCs and TNRC6 proteins. (**A**) Human TNRC6 proteins (yellow) have three AGO-binding sites, Motifs I and II, and AGO Hook. Each binding site includes at least two tryptophan residues shown as ‘W’ in a circle. The three tryptophan-binding sites on RISC are depicted as white circles. (**B**) A TNRC6 protein (yellow) binds to two RISCs loaded with different miRNAs (red and magenta). These RISCs cooperatively bind to a target mRNA that includes their binding sites. (**C**) Model of increasing the local concentration of mRNA. A TNRC6 protein (yellow) binds to three RISCs loaded with different miRNAs (blue, red, and magenta). Two RISCs on the left and right sides interact with mRNA1 and mRNA3 in guide-dependent manners. Another RISC in the middle touches, through the surface, mRNA2 in a guide-independent manner. Guide-independent interactions between RISCs and mRNAs are depicted as black arrows. (**D**) Tryptophan-binding pockets on the exterior of the AGO4 PIWI domain (PDB ID: 6OON). The domain color codes are the same as in Figure 3B.

The N-terminal region of TNRC6 proteins is known as the AGO-binding domain (ABD) consisting of three AGO-binding sites, each of which includes at least two tryptophan (Trp) residues that are separated by ten or more amino acids (Figure 14A) (102, 103). On the other hand, RISCs have three Trp-binding pockets on the exterior of the PIWI domain (Figure 14A) (16, 104). A crystal structure revealed that a TNRC6 fragment inserts its two Trp residues into the two out of three Trp-binding pockets (101). This study also showed 2D class average of the cryo-electron microscopy density map that three RISCs bound to the ABD of the same TNRC6 protein. Therefore, one TNRC6 protein can interact with up to three RISCs simultaneously. Briskin et al. reported that two RISCs loaded with different miRNAs connected by TNRC6 protein cooperatively bind to their binding sites on the same target mRNA (Figure 14B) (105). Given that RISCs keep binding to different mRNAs transiently through the exterior, the three-way interactions among RISC, TNRC6, and mRNA would drastically increase the local concentration of mRNAs (Figure 14C), which would enhance the opportunity to meet many different mRNAs in a short time and thus benefit the first step of target recognition by RISCs (Figure 12B).

Four human AGOs are co-immunoprecipitated with TNRC6A, 6B and 6C in HEK293 cells (106), whereas all paralogs, but not AGO4, are colocalized with TNRC6A in P-bodies of HeLa cells (107). AGO1, AGO2 and AGO3 share exactly the same three Trp-binding pockets, Pocket-1, -2 and -3, on the exterior of the PIWI-catalytic subdomain (104). Although the Pocket-1 of AGO4 is identical, the Pocket-2 and -3 are slightly different from those of the other paralogs (Figure 14D) (15). The different affinity of AGO4 for TNRC6 proteins in particular cells or environments may be attributed to its unique Trp-binding Pocket-2 and -3.

## IMPACT OF POST-TRANSLATIONAL MODIFICATION ON THE TARGET SPECIFICITY

Previous studies reported that human AGOs undergo post-translational modifications (PTMs), such as phosphorylation (31, 108, 109), sumoylation (110, 111), acetylation (112), ubiquitination (113), prolyl-4-hydroxylation (114), and poly-ADP-ribosylation (115). This section will describe how those PTMs modulate the activities of AGOs.

### Phosphorylation changes the affinity of RISC for mRNAs

**Figure 15.**
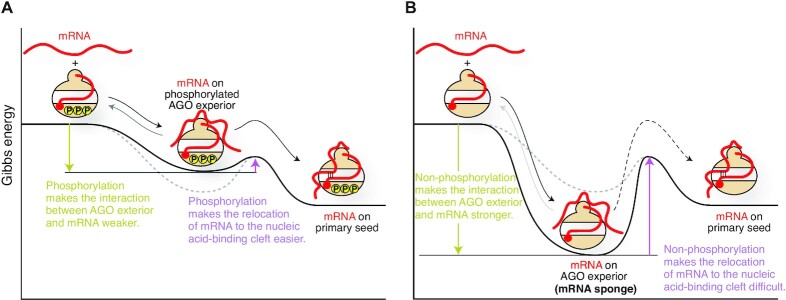
Impact of the phosphorylation of AGO on target binding. The energy levels of hyperphosphorylated AGO (**A**) and non-phosphorylated AGO (**B**) are shown as reaction coordinates.

The phosphorylation of a C-terminal serine cluster (S824–S834 in the case of AGO2), located on Loop 5 in the PIWI-helical subdomain (Figure 3A), enhances the release of the bound mRNA (31, 116). AGO mutants deficient in phosphorylation at the cluster increased association with target mRNAs (116). Reversible phosphorylation would manipulate the Gibbs free energy of ‘mRNA-bound RISC’. The phosphorylation of the C-terminal serine cluster would make the interaction between mRNA and RISC exterior thermodynamically unfavored (Figure 15A). The weakened interaction would let the mRNAs leave unless they have a complementary sequence, which increases the turnover of mRNA binding. As a result, a phosphorylated RISC can find a complementary target more quickly and thus facilitates gene silencing. The degree of phosphorylation at the C-terminal serine cluster could regulate the speed of the gene silencing.

When the serine cluster is not phosphorylated at all, mRNAs remain bound quite stably to the RISC exterior. In this case, the Gibbs free energy of ‘mRNA-bound RISC’ would be lower than that of the phosphorylated state, and thus result in an increased energy barrier to move on to the state of ‘mRNA pairing with the primary seed’ (Figure 15B). Such a stable interaction is expected to interfere with the mRNA relocation from the surface to the nucleic acid-binding cleft and slows down the release of the mRNA to solvent. As a result, the dephosphorylated RISC cannot cause gene silencing and probably works as an mRNA sponge.

### AGO2–AGO3 switching

LIM Domain containing 1 (LIMD1), Ajuba, and Wilms Tumor-Interacting Protein 1 (WTIP) were identified as essential components for miRNA-mediated, but not siRNA-mediated, gene silencing (117). The follow-up study revealed that the phosphorylation of AGO2 Ser387 by AKT Serine/Threonine Kinase 3 (Akt3), one of three AKT kinases, is essential to recruit LIMD1 to AGO2 and that their complex formation facilitates interaction with TNRC6A and DDX6, the latter of which is recruited by the CCR4/NOT complex (107, 118, 119). Akt3-mediated phosphorylation downregulates target mRNA cleavage and upregulates translational repression (120). Ser387 is located on the exterior of the N-PAZ lobe while the TNRC6 protein-binding sites (i.e. the Trp-binding pockets) are on the MID-PIWI lobe. Their relative positions suggest that the binding of LIMD1 to the AGO2 N-PAZ lobe forms a composite scaffold to interact with TNRC6A. Since AGO1 and AGO4 also retain the corresponding Ser (Figures 9D and 10), their TNRC6A-mediated gene silencing must be similarly regulated by Akt3. In contrast, AGO3 lacks the serine, but possesses a glutamate in the vicinity that mimics a phosphorylated serine and thus can always interact with LIMD1, Ajuba and WTIP, independently of Akt3. Upon LIMD1 ablation in HeLa cells, the main effector complex switches from AGO2-LIMD1 to AGO3-WTIP. Although both AGO2 and AGO3 bind to WTIP and share the same TNRC6-binding pockets, only AGO3–WTIP complex can recruit TNRC6A efficiently (107).

During differentiation from embryonic stem cells (ESC) to mesenchymal stem/stromal cells, the protein level of AGO3 showed a steep increase and become more abundant than AGO1 and AGO2 (121). This AGO2-to-AGO3 switch happens with an increase in the protein level of LIMD1, which would make more AGO3-LIMD complexes that facilitate TNRC6-mediated gene silencing independently of Akt3. The significance of AGO3 when ESCs lose pluripotency was also reported in all-trans retinoic acid-induced neural development (8).

### Sumoylation/ubiquitination

K400 of AGO1 is sumoylated, which enhances miRNA activity (122). AGO2 would follow the same mechanism because the corresponding K402 undergoes sumoylation (110). On the other hand, neither AGO3 nor AGO4 has the corresponding lysine (Figures 9D and 10). Mutation of K402 made the half-life of AGO2 longer, suggesting that sumoylation at K402 by SUMO1 and SUMO3/4 destabilizes AGO2 (110). These lysine residues are located on the exterior of the L2 domain. Recent studies reported that TDMD is required for ubiquitination of specific lysine residues, which do not include K400 or K402 (85,86).

### Nitrosylation and hydroxyproline

C691 and P700 of AGO2 are conserved across the four AGOs (Figures 9D and 10) and participate in forming Trp-binding Pocket-3 (Figure 14D). C691 of AGO2 undergoes S-nitrosylation to reduce affinity for TNRC6 protein (123). Prolyl 4-hydroxylase modifies the corresponding proline residue in AGO2 and AGO4 more efficiently than that in AGO1 and AGO3, increasing the stability of AGO2 (114). It remains to be studied whether the generated hydroxyproline affects the interaction of the AGO with TNRC6 proteins.

## DELETIONS AND MUTATIONS POSSIBLY RELEVANT TO DISEASES

### C-terminal extended AGO1, AGO1x

**Figure 16.**
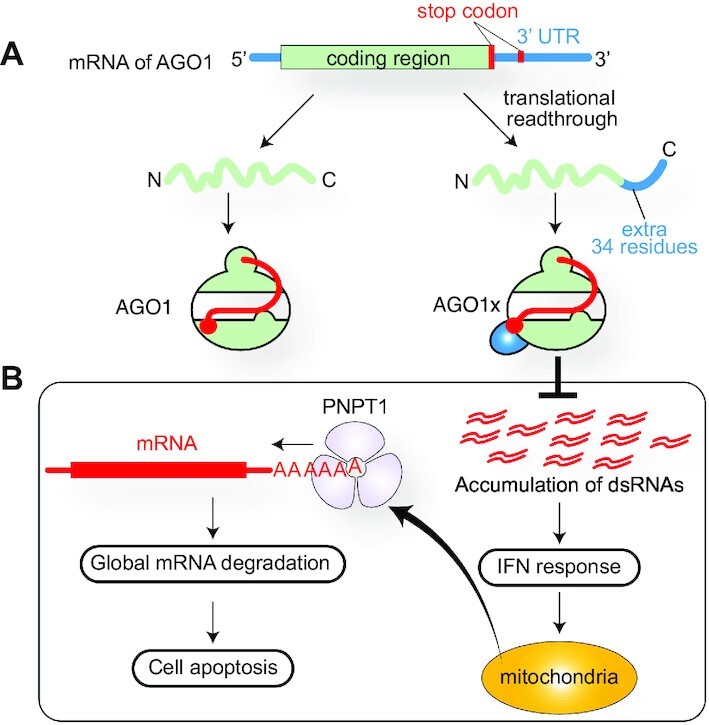
Synthesis and role of AGO1x. (**A**) Translational readthrough of the AGO1 mRNA generates AGO1x. (**B**) AGO1x inhibits the interferon response and prevents apoptosis.

A genome-wide analysis identified that the AGO1 mRNA includes a let-7a miRNA-binding site downstream of the canonical AGO1 stop codon (124). Binding of let-7a-loaded AGO to the site causes a translational readthrough, generating a C-terminal extended AGO1 isoform named AGO1x (Figure 16A) (125). The extra region contains 34 amino acids. Canonical AGO1, as well as the other paralogs, uses its C-terminal carboxyl group to recognize the 5′ monophosphate group of guide RNA through water molecules (Figure 3D) (13, 18). Due to its extended C-terminus, AGO1x would not form the composite 5′-end binding site, whose formation is essential for interaction with TNRC6 proteins (15). As a result, AGO1x loads miRNAs differently and does not cause post-transcriptional gene silencing (PTGS) because of failure to interact with TNRC6 proteins (125). Instead, AGO1x competes with the canonical AGO1 for target mRNAs, thereby reducing the gene silencing. AGO1x, but not AGO1, interacts with polyribonucleotide nucleotidyltransferase 1 (PNPT1), which is a 3′→5′ exoribonuclease that initiates global mRNA degradation and thus causes cell apoptosis (Figure 16B). AGO1x-depleted cells accumulate double-stranded RNAs, which activate the interferon response and make the cells more resistant to virus infection (126). This study also reported that the expression of AGO1x in breast cancer patients was positively correlated with that of Ki-67, a proliferation marker for human tumor cells.

High nucleotide conservation after the stop codon across vertebrates is the hallmark of translational readthrough (126). Similar conservations are also seen in the other AGO paralogs (125), suggesting the possibility that the other three paralogs generate a C-terminal extended isoform, too.

### Deletions and microdeletions

The genes of AGO4, AGO1 and AGO3 reside in tandem on human chromosome 1 in this order. Microdeletions of the chromosomal region encoding AGO1 and AGO3 genes were found in five patients with hypotonia, poor feeding, and developmental delay, suggesting the possibility that AGO1 and AGO3 are involved in neurocognitive deficits (12). Chromosome microarray studies identified 10 individuals with a 2.3 Mb deletion including AGO1 and AGO3. The patients with haploinsufficiency suffered from global developmental delay, mild intellectual disability, delayed bone age, and so on (127).

### Point mutations

**Figure 17.**
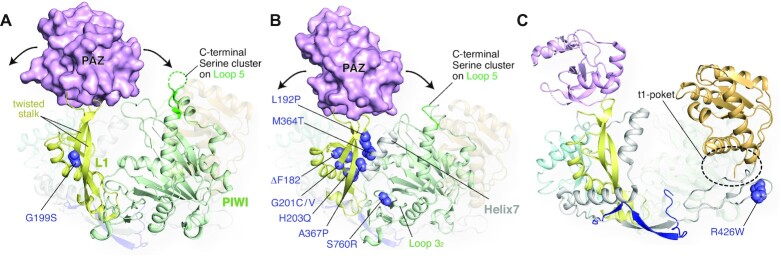
Mutations possibly relevant to neural diseases. (**A**) AGO1 G199S mutation would break the twisted stalk in the L1 domain and affect the relative position of the PAZ domain against Loop 5. Part of the AGO1 structure (PDB ID: 4KXT) is transparent for clarity. (**B**) Many AGO2 mutations found in neural disease patients are located on the stalk in the L1 and Helix 7 in the L2 domain. (**C**) AGO3 R426W mutation, found in obsessive-compulsive disorder, seems to affect the t1 pocket.

AGO1 mutations have been identified in patients with intellectual disability or autism spectrum disorder (11, 128–132). In addition, several studies reported typical facial features among patients with intellectual disabilities whose genomes have a mutation on the AGO1 gene (11, 12, 132). These results raise a possible correlation between the AGO1 mutations and the syndromes of intellectual disability. For instance, Hamdan et al. reported a missense mutation of Gly199 to Ser (130) as a possibly intellectual disability-related de novo mutation. The same mutation was also found by a whole-exome analysis of a proposita, who had diffuse hypotonia, infrequent seizures, and an intellectual disability with an intelligence quotient of 41 (11). This glycine is located in the middle of one of the two long, twisted antiparallel β-strands that serve as a ‘stalk’ to hold the PAZ domain above the crescent of AGO (Figure 17A) (26). In the AGO1 structure, Gly199 takes the dihedral angles, *φ* = –167.89 and *ψ* = –150.83 (13), which is allowed for only glycine due to the lack of C*β*. Therefore, Gly199 plays the essential role in twisting the stalk uniquely, and the mutation would change the position of the PAZ domain against the rest of the AGO.

A recent study reported 13 germline AGO2 mutants, including Gly201 corresponding to the Gly199 of AGO1, possibly relevant to neurological development (133). Interestingly, most mutants (Phe182 deletion, Leu192Pro, Gly201Cys, His203Gln, Met364Thr, Ala367Pro, and Ser760Arg) retained the abilities of miRNA loading, target cleavage, gene silencing and binding to TNRC6, similarly to the wild type (133). These AGO2 mutants, except for His203Gln, had a less phosphorylated C-terminal serine cluster and bound more mRNAs compared to the wild type. The authors pointed out the correlation in disease-causing AGO2 mutants between the reduced phosphorylation level of the C-terminal serine cluster on Loop 5 and the extended dwelling time with target mRNAs (133). Since these mutation sites are located on the stalk and Loop 3_2_, their mutants seem to change the relative position between the PAZ domain and the C-terminal serine cluster (Figure 17B). In those mutants, the PAZ domain may be placed improperly so that the serine cluster is less accessible to casein kinase α1. Interestingly, the serine cluster remained unmodified in miRNA-binding deficient AGO2 mutants (31). Given that AGOs drastically change the conformation before and after incorporating a miRNA (15), the kinases may recognize the typical structural features that only the bilobed RISC has.

Meanwhile, ANKRD52-PPP6C phosphatase complex and CSNK1A1 kinase compete for the serine cluster on Loop 5 to control the phosphorylation level, and AGO2 with non-phosphorylation can expand the target repertoire (i.e. more off-target interactions) (116). These facts would suggest the following hypothesis: wild-type AGOs, whose serine cluster is phosphorylated to some extent, catch and release many mRNAs quickly to achieve ‘on-target’ interactions. However, the de novo mutations relevant to neural diseases elongate the dwelling time between their RISCs and mRNAs, drastically reducing opportunities to meet many mRNAs. Even worse, the extended dwelling time would allow the AGO mutants to initiate translational repression or degradation of ‘off-target mRNAs’. The resultant dysregulation of gene expression would cause neural diseases.

A recent study analyzed a whole-genome sequencing on 53 parent-offspring families with offspring affected by obsessive-compulsive disorders and reported a mutation of cytidine to thymidine at a position of 1:36479519 as a de novo mutation possibly relevant to obsessive-compulsive disorder (134). The paper reported that the mutation changes Arg192 to Trp. This residue number was counted from the N-terminus of an AGO3 isoform in which the N-terminal 234 amino acids of the full-length AGO3 is missing. Therefore, Arg192 of the short AGO3 isoform corresponds to Arg426 on the L2 domain of the full-length AGO3 (Figure 17C). The mutation to Pro seems to affect the formation of the t1 pocket (135).

Lastly, it is intriguing that most of the reported disease-relevant mutations are sporadic and heterozygous, which prompted me to think about the possibility that their homozygous mutations become lethal and therefore have not been detected.

## SUMMARY

Accumulated evidence indicates that, presumably, the four human AGOs work redundantly when cells simply need to maintain homeostasis. In this case, even if one of the four AGOs cannot work properly, the other paralogs would compensate. Supporting this idea, mouse ESCs died if all four AGOs were defective, but were rescued by reintroducing any single AGO (136). However, when cells drastically change their environments, such as in neural development or viral infection, a particular type of AGO loaded with a specific miRNA is required to execute specialized commands. This could explain why patients lacking a functional AGO can be alive, yet suffer from autism spectrum disorder, intellectual disability, or obsessive-compulsive disorder due to dysregulation of proper neural development. Therefore, it is expected that understanding the specialized role of each AGO paralog is vital to developing the next generation of RNAi therapeutics and clinical research.

The crystal structures of RISCs revealed that the topology of AGO within the RISC is extremely sophisticated. The polypeptide of AGO travels between the two lobes, back and forth, while the loops protruding from different domains interact directly or indirectly via the bound guide RNA and trapped water molecules (15). This complicated topology makes it challenging to design stable AGO mutants, especially constructs in which a specific domain is deleted. For example, the fusion of a piece of Beam to the MID domain was necessary to make an isolated MID-PIWI lobe stable (61). Thus far, many domain-deleted AGO mutants have been used to test whether the resultant construct retains the physiological function. Many constructs, however, seem to be designed without consideration of the tertiary structure, which reminds me of a message in a recent review paper, blowing the whistle on research lacking in rigor (137). Since the atomic-resolution crystal structures of four human AGOs are available now, future research can exploit their structural information to design the most appropriate constructs for their experiments. Using carefully designed constructs will enable us to tackle the remaining enigmatic questions and pursue exciting studies on AGOs and small non-coding RNAs.

## Supplementary Material

gkac519_Supplemental_Files
