# Supplementary material for: Anatomy of four human Argonaute proteins
Source: Nucleic Acids Res. 2022 Jun 23;50(12):6618–38. doi: 10.1093/nar/gkac519 (PMC9262622; doi:10.1093/nar/gkac519)
Supplement: gkac519_Supplemental_Files [file gkac519_supplemental_files.zip › Supplementary-Movie-1_legend.docx]

**Supplementary Movie 1.** The electrostatic potential of AGO2 in complex with a guide and a target (PDB ID: 6NO4). The target strand is removed to show the guide-contacting area.
